# Efficacy and safety of ruxolitinib combined with steroids for first-line treatment of acute graft-versus-host disease after hematopoietic stem cell transplantation: a single-center, real-world experience

**DOI:** 10.3389/fimmu.2025.1621708

**Published:** 2025-07-14

**Authors:** Yan Yang, Yuan Huo, Dong Zhou, Zhijie Kang, Yanan Huang, Ying Wang, Guangjun Fan, Liyuan Ma, Jinsong Yan

**Affiliations:** ^1^ Department of Hematology, Liaoning Medical Center for Hematopoietic Stem Cell Transplantation, Second Hospital of Dalian Medical University, Dalian, China; ^2^ Department of Pharmacy, Second Affiliated Hospital of Dalian Medical University, Dalian, China; ^3^ Department of Hematology, Shanghai Ninth People’s Hospital Affiliated to Shanghai Jiaotong University School of Medicine, Shanghai, China; ^4^ Liaoning Key Laboratory of Hematopoietic Stem Cell Transplantation and Translational Medicine, Dalian Key Laboratory of Hematology, Diamond Bay Institute of Hematology, Blood Stem Cell Transplantation Institute, Second Hospital of Dalian Medical University, Dalian, China; ^5^ Department of Hematology, Yingkou People’s Hospital, Yingkou, Liaoning, China

**Keywords:** graft-versus-host disease, hematopoietic stem cell transplantation, ruxolitinib, steroids, leukemia

## Abstract

**Introduction:**

Despite the increasing use of allogeneic hematopoietic stem cell transplantation (allo-HSCT), graft-versus-host disease (GVHD) remains the main cause of morbidity and mortality, significantly impacting HSCT outcomes. Steroids are the standard first-line treatment for acute GVHD (aGVHD); however, standardized treatment algorithms for patients who do not respond to steroid therapy are lacking. Ruxolitinib is the most promising second-line therapy for steroid-refractory (SR)-GVHD, but data on its first-line use for aGVHD are limited.

**Methods:**

In this retrospective study, we analyzed the data of 133 patients with aGVHD who underwent transplantation at our institution. Eighty-three patients received ruxolitinib combined with methylprednisolone, while 50 received methylprednisolone alone as the initial treatment.

**Results:**

The ruxolitinib/steroids group had a significantly higher overall response rate (ORR) on day 7 (86%) compared to the steroid-only group (68%; odds ratio [OR]=2.8, 95% confidence interval [CI]: 1.2–6.5, p=0.019). Similarly, ORR on day 14 was higher in the ruxolitinib/steroids group (92% vs. 79%; OR=2.7, 95% CI: 0.9–7.8, p=0.05). Although no statistical differences were observed in overall survival (OS), progression-free survival (PFS), and failure-free survival (FFS) between the two groups, patients who achieved early ORR on days 7 and 14 had better OS, PFS, and FFS. Additionally, in subgroup analysis of patients who underwent peripheral blood stem cell transplantations, the ruxolitinib/steroids cohort had significantly better OS (Hazard Ratio [HR]=0.34, 95% CI: 0.11–1.55, p=0.04), PFS (HR=0.37; 95% CI: 0.12–1.10, p=0.05) and FFS (HR=0.46; 95% CI: 0.19–1.11, p=0.05) compared to the steroid-only cohort. Adverse event (AEs) frequencies were comparable between groups, with the exception of neutropenia (32.5% vs. 12%, p=0.008) and CMV infection (34.9% vs. 18%, p=0.036), which were more frequent in the ruxolitinib/steroid group.

**Discussion:**

To the best of our knowledge, this is the first real-world study to demonstrate that adding ruxolitinib to a standard methylprednisolone regimen provides an effective and safe first-line treatment for aGVHD.

## 1 Introduction

Allogeneic hematopoietic stem cell transplantation (allo-HSCT) is a vital curative treatment modality for various malignant and benign hematologic diseases. Acute myeloid leukemia, myelodysplastic syndrome/myeloproliferative disorders, and acute lymphoblastic leukemia are the most common indications for allo-HSCT (1–3). Acute graft-versus-host disease (aGVHD) develops in 50–70% of patients following allo-HSCT with conventional prophylaxis and is a major cause of non-relapse mortality (NRM) in patients receiving transplantation, posing a significant challenge to successful transplant outcomes (4, 5).

The etiology of aGVHD is complex, and its pathophysiology can be divided into three sequential phases: (I) activation of host antigen-presenting cells (APCs); (II) activation of donor T cells, leading to their clonal expansion and differentiation; and (III) destruction of target tissue by inflammatory effector cells and cytokines, affecting multiple organs, particularly the skin, gastrointestinal tract, and liver (6–9). Human leukocyte antigen (HLA) mismatch represents the most important risk factor for aGVHD. Other risk factors include donor-recipient sex disparity, conditioning regimen intensity, underlying disease, aging, multiparous female donors, graft source, and insufficient GVHD prophylaxis (10, 11). The standard first-line treatment for aGVHD is high-dose glucocorticoids (12–14). However, approximately 50% of patients become steroid-resistant or refractory (SR), resulting in poor long-term prognosis, with an estimated NRM rate of 40% within 12 months (15, 16). To date, no consensus has been established regarding the optimal management of SR-aGVHD; however, the FDA approved ruxolitinib for SR-aGVHD in 2019, marking a significant step toward standardizing its initial treatment (17).

Ruxolitinib is an orally administered selective inhibitor of Janus kinase (JAK)1/2. JAKs are intracellular tyrosine kinases, crucial for the development and function of immune cells, and are implicated in aGVHD pathogenesis (18). Ruxolitinib-induced immunomodulation is hypothesized to involve reduced neutrophil migration during the first phase of aGVHD, decreased T-cell priming via MHC-II downregulation, reduced cytokine release during the second phase, and limited T-cell expansion in the third phase (19). Several retrospective clinical studies of ruxolitinib, as salvage therapy for SR-aGVHD, have demonstrated its clinical benefits (20, 21). The FDA approval of ruxolitinib for SR-aGVHD was based on the results of REACH1, an open-label, single-arm phase II trial that enrolled subjects from December 2016 to July 2018 (17, 22).

Studies on ruxolitinib as a first-line treatment for aGVHD are limited. Researchers from China performed a multicenter, randomized, phase III trial to evaluate the efficacy and safety of ruxolitinib plus steroids for aGVHD. The study demonstrated that adding ruxolitinib to the standard methylprednisolone regimen provided an effective and safe first-line treatment for newly diagnosed high-risk aGVHD (23). We conducted a retrospective, real-world study to investigate the therapeutic efficacy and safety of ruxolitinib combined with methylprednisolone as a first-line treatment for aGVHD.

## 2 Materials and methods

### 2.1 Patients

A retrospective analysis was conducted to evaluate the treatment outcomes of 133 patients who underwent allo-HSCT and were diagnosed with aGVHD at our center between August 2014 and June 2023. The underlying diseases included aplastic anemia (AA), acute myeloid leukemia (AML), acute lymphocytic leukemia (ALL), myelodysplastic syndrome (MDS), and congenital hemophagocytic syndrome. The marrow transplant databases of the Second Affiliated Hospital of Dalian Medical University in China were screened for aGVHD after allo-HSCT diagnosis. The patient outcomes were monitored until the end of June 2024. This study was approved by the Ethics Committee of the Second Affiliated Hospital of Dalian Medical University.

### 2.2 Allo-HSCT procedure

All 133 patients underwent myeloablative (MA) conditioning regimen transplantation, except for those with AA. This included the treatment of acute leukemia and MDS based on the Beijing protocol, which utilized a modified BU/CY conditioning regimen as follows: cytarabine (2 g/m^2^ q12h for 2 days; qd for fully matched donors), busulfan (3.2 mg/kg/day for 4 days), cyclophosphamide (1.8 g/m^2^ for 3 days), and anti-thymocyte globulin (ATG) (2.5 mg/kg/day for 4 days; 2 days for fully matched donors) (24). Enhanced MA regimens, including decitabine, idarubicin, and clarithromycin, were administered according to the risk of primary disease and disease status before transplantation (25). For AA, the FLU/CY conditioning regimen, combined with ATG to suppress T cells, involved the administration of 30 mg/m^2^ fludarabine for 4 days, 30–50 mg/kg cyclophosphamide for 2 days, and 2.5 mg/kg/d ATG for 4 days (26). Most transplantation recipients received ATG, cyclosporine A (CsA)/Tacrolimus (FK506), mycophenolate mofetil, and short-term methotrexate for GVHD prophylaxis.

### 2.3 Study design

The grading and staging systems of aGVHD were based on the Glucksberg or Mount Sinai aGVHD International Consortium (MAGIC) criteria (27, 28). Individuals were divided into two treatment groups. In the ruxolitinib/steroids combination group, patients were given methylprednisolone at a dose of 0.5–2 mg/kg/day, and ruxolitinib was administered orally at a daily dose of 10–15 mg. If patients with aGVHD responded to treatment by achieving partial response (PR) or complete response (CR) within 7 days, the methylprednisolone dosage was gradually tapered. If GVHD did not recur after steroid discontinuation, the ruxolitinib dose was gradually reduced. In the steroid-only group, patients were given methylprednisolone at a dose of 0.5–2 mg/kg/day, which was gradually tapered and discontinued after CR. The initial steroid dosage for both groups was determined based on the severity of the aGVHD. For mild patients (grade I and II aGVHD, only involving the upper digestive tract), the initial dosage was 0.5 mg/kg. For grade III/IV aGVHD involving the gastrointestinal tract or hyperacuteGVHD, the initial dosage of methylprednisolone was 2 mg/kg. For most grade II-III aGVHD, the dosage was 1 mg/kg. The steroid dosage in both groups were basically balanced. According to guidelines and relevant literature (12), during the treatment of aGVHD, both groups of CNI were adjusted to effective therapeutic concentrations (cyclosporine trough concentration to 150-250ng/ml, FK506 5-15ng/ml). After aGVHD achieves CR, stop steroid first, and then tappered gradually and eventually discontinued CNI. The CNI reduction plan for both groups is the same. Second-line therapy was initiated in both groups for patients with refractory aGVHD, defined as GVHD progression after 3 days of treatment, lack of improvement within 7 days, or failure to achieve CR after 14 days.

### 2.4 Endpoints

The primary endpoint was the overall response rate (ORR) to aGVHD treatment at 3, 7, 14, and 28 days post-intervention. ORR was defined as the percentage of patients in each group who attained either PR or CR without requiring additional immunosuppressive agents. CR was characterized by the complete resolution of aGVHD symptoms, whereas PR was defined as an improvement in at least one stage in a single organ without worsening in others. No response (NR) was classified as no improvement, worsening symptoms in any organ, or the emergence of new GVHD-related symptoms. Additionally, GVHD progression after 3 days of therapy or lack of improvement within 7 days was considered NR. Secondary endpoints included overall survival (OS), progression-free survival (PFS), failure-free survival (FFS), cumulative incidence of relapse (CIR), NRM, and safety. OS was defined as the time from aGVHD onset to death from any cause. PFS was defined as the time from aGVHD onset to relapse of the primary disease or death from any cause, whichever occurred first. Failure-free survival (FFS) refers to the time from aGVHD onset to disease relapse or progression, NRM, or the initiation of additional therapy for aGVHD. Safety endpoints were assessed based on the frequency of adverse events (AEs), defined according to the National Cancer Institute Common Terminology Criteria for Adverse Events (NCI-CTCAE 4.0).

### 2.5 Statistical analyses

Data were analyzed using STATA/SE 15.1 software (STATA Corp, College Station, Texas, USA). ORR, CR, and PR were analyzed using Pearson’s chi-square test. The correlation between ORR and different independent variables was calculated using regression analysis. The Kaplan-Meier method was used to estimate OS, PFS and FFS, and the Log-rank test was used to determine statistical significance. NRM and cumulative incidence of relapse were estimated by considering each other as competing risks. The reverse Kaplan-Meier method was used to calculate the median follow-up duration. Prognostic variables for OS were evaluated by univariate and multivariate analyses using Cox proportional hazard regression. Variables with statistical significance (p <0.1) in the univariate analysis were included in the multivariate analysis to adjust for potential confounding effects. A p-value <0.05 was considered statistically significant. The following variables were evaluated: sex, age at allo-HSCT (<60 years vs. ≥60 years), donor sex (female donor to male recipient vs. others), disease type (malignant vs. benign), donor type (cord blood, CBT vs. haplo-identical donor, HID vs. matched sibling donor, MSD vs. unrelated donor, URD), HLA type (fully match vs. haplo), blood type (match vs. mismatch), graft origin (CBT vs. PB+BM+CB vs. PB+BM vs. PB), hematopoietic cell transplant-comorbidity index (HCT-CI: 2 vs. 1 vs. 0), aGVHD grade (IV vs. III vs. II vs. I), aGVHD skin involvement (yes vs. no), aGVHD GI involvement (yes vs. no), aGVHD liver involvement (yes vs. no), day 3 ORR (yes vs. no), day 7 ORR (yes vs. no), day 14 ORR (yes vs. no), and day 28 ORR (yes vs. no). Pearson’s chi-square test was used to analyze AEs between different groups, including neutropenia, thrombocytopenia, cytomegalovirus (CMV) infection, Epstein-Barr virus (EBV) infection, renal toxicity, cardiac toxicity, digestive reactions, nerve toxicity, electrolyte disturbance, blood glucose disturbance, blood lipid disturbance, and hypertension.

## 3 Results

### 3.1 Patient and treatment characteristics

The patient demographics and baseline disease characteristics are presented in **Table 1**. This study included a cohort of 133 patients with aGVHD after allo-HSCT. Of these, 83 patients were classified into the ruxolitinib/steroid group, while 50 patients were in steroid-only group. The overall median age was 30 (0.5–67) years in the ruxolitinib/steroid group and 27.5 (1–61) years in the steroid-only group. The median body weight was 55 (9–100) kg vs. 52 (8.1–97) kg, respectively. The median number of infused mononuclear cells (MNC) was 9.41 × 10^8/kg (range: 4.68–20.4) in the ruxolitinib/steroid group and 10.31×10^8/kg (range: 4.27–28.3) in the steroid-only group. The median number of infused CD34+ cells was 6.63×10^6/kg (range: 2.29–22.7) and 6.01×10^6/kg (range: 0.99–21.7), respectively. The baseline demographic, transplantation-related, and disease-related patient characteristics-including recipient sex, disease type, donor type, donor sex, stem cell source, conditioning regimen intensity, recipient/donor blood type pair, GVHD prophylaxis, ATG use, HCI-CI, aGVHD skin involvement, aGVHD GI involvement, and aGVHD liver involvement-were comparable between the two groups. All patients underwent neutrophil engraftment, with no significant difference in the time to neutrophil or platelet engraftment between the two groups. Notably, grade I aGVHD was more common in the steroid-only group, while grade II aGVHD was more frequent in the ruxolitinib/steroids group (p=0.008).

**Table 1:** Baseline patient characteristics (n=133).

Characteristic	No. (%)	No. (%)	p value
	R+S (n=83)	S (n=50)	
Age, median (range), y	30 (0.5–67)	27.5 (1–61)	
Weight, median (range), kg	55 (9–100)	52 (8.1–97)	
Sex			0.912
Male	44 (53)	27 (54)	
Female	39 (47)	23 (46)	
Disease type			0.685
Benign	19 (22.9)	13 (26)	
Malignant	64 (77.1)	37 (74)	
Donor type			0.1
HID	62 (74.7)	29 (58)	
MSD	19 (22.9)	21 (42)	
URD	1 (1.2)	0 (0)	
CBT	1 (1.2)	0 (0)	
Donor gender			0.609
Female to male	18 (21.7)	9 (18)	
Others	65 (78.3)	41 (82)	
Stem cell source			0.527
PB	14 (16.9)	11 (22)	
BM+PB	58 (69.9)	36 (72)	
BM+PB+CB	10 (12)	3 (6)	
CB	1 (1.2)	0 (0)	
Conditioning regimen			
MAC	83 (100)	50 (100)	0.707
RIC/NMA	0 (0)	0 (0)	
GVHD prophylaxis			0.0001
CSA based	1 (1.2)	21 (42)	
FK506 based	82 (98.8)	29 (58)	
Anti-thymocyte globulin			0.797
Yes	82 (98.8)	50 (100)	
No	1 (1.2)	0 (0)	
Blood type			0.205
Match	54 (65)	27 (54)	
Mismatch	29 (35)	23 (46)	
HCT-CI			0.426
0	25 (30.1)	14 (28)	
1	58 (69.9)	35 (70)	
2	0 (0)	1 (2)	
aGVHD grade			0.008
I	16 (19.3)	21 (42)	
II	52 (62.7)	17 (34)	
III	8 (9.6)	8 (16)	
IV	7 (8.4)	4 (8)	
aGVHD skin involvoment			0.122
Yes	40 (48.2)	31 (62)	
No	43 (51.8)	19 (38)	
aGVHD GI involvoment			0.383
Yes	56 (67.5)	30 (60)	
No	27 (32.5)	20 (40)	
aGVHD liver involvement			0.061
Yes	9 (10.8)	1 (2)	
No	74 (89.2)	49 (98)	
MNC, median (range)			
10^8/kg	9.41 (4.68–20.4)	10.31 (4.27–28.3)	
CD34+, median (range)			
10^6/kg	6.63 (2.29–22.7)	6.01 (0.99–21.7)	
Steroid dosage			0.11
0.5mg/kg	43	17	
1mg/kg	33	26	
2mg/kg	7	7	

R, ruxolitinib; S, steroid; HID, haploidentical donor; MSD, matched sibling donor; URD, matched unrelated donor; CBT, cord blood transplantation; BM, bone marrow; PB, peripheral blood; CB, cord blood; MAC, myeloablative conditioning; RIC, reduced-intensity conditioning; NMA, non-myeloablative; CSA, cyclosporine A; FK506, tacrolimus; HCT-CI, hematopoietic cell transplant-comorbidity index; aGVHD, acute graft versus host disease; MNC, mononuclear cell.

### 3.2 Primary endpoint

**Figure 1:**
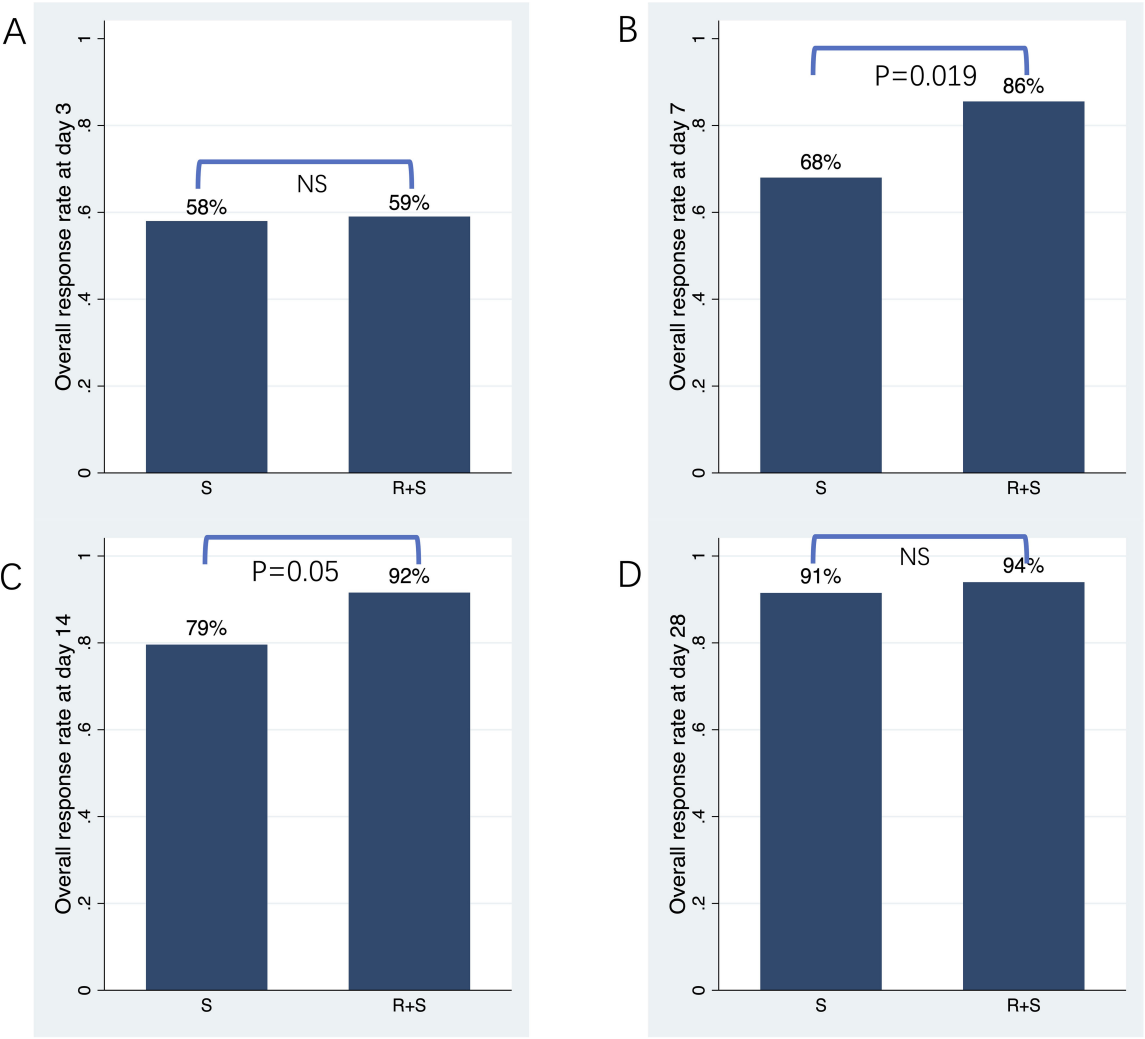
Overall response rate on days 3, 7, 14 and 28 post-treatment. **(A)** Overall response rate on day 3. **(B)** Overall response rate on day 7. **(C)** Overall response rate on day 14. **(D)** Overall response rate on day 28.

The primary endpoint was ORR at 3, 7, 14 and 28 days post-intervention. As shown in **Figure 1A** and **Table 2**, ORR on day 3 was comparable between the two groups (59% vs 58%; odds ratio [OR]=1.04, 95% confidence interval [CI]: 0.51–2.13, p=0.906). On day 7, ORR was significantly higher in the ruxolitinib/steroids combination group (86%) compared to the steroid-only group (68%; OR=2.78, 95% CI: 1.18–6.53 p=0.019; **Figure 1B**, **Table 2**). Similarly, ORR on day 14 was significantly greater in the ruxolitinib/steroids combination group (92%) compared to the steroid-only group (79%; OR=2.77, 95% CI: 0.98–7.87, p=0.05; **Figure 1C**, **Table 2**). The ruxolitinib/steroids combination cohort showed similar ORR on day 28 compared with the steroid-only cohort (94% vs 91%; OR=1.45, 95% CI: 0.37–5.69, p=0.593; **Figure 1D**, **Table 2**).

**Table 2:** Response assessment post treatment.

ORR	R+S (n=83)	S (n=50)	Odds ratio (95% CI)	*p*
Day 3 ORR	59% (49/83)	58% (29/50)	1.04 (0.51–2.13)	0.906
Day 7 ORR	86% (71/83)	68% (34/50)	2.78 (1.18–6.53)	0.019
Day 14 ORR	92% (76/83)	79% (39/49)	2.77 (0.98–7.87)	0.05
Day 28 ORR	94% (78/83)	91% (43/47)	1.45 (0.37–5.69)	0.593

ORR, overall response rate; R, ruxolitinib; S, steroid; 95% CI, 95% confidence interval.

### 3.3 Secondary endpoint

**Figure 2:**
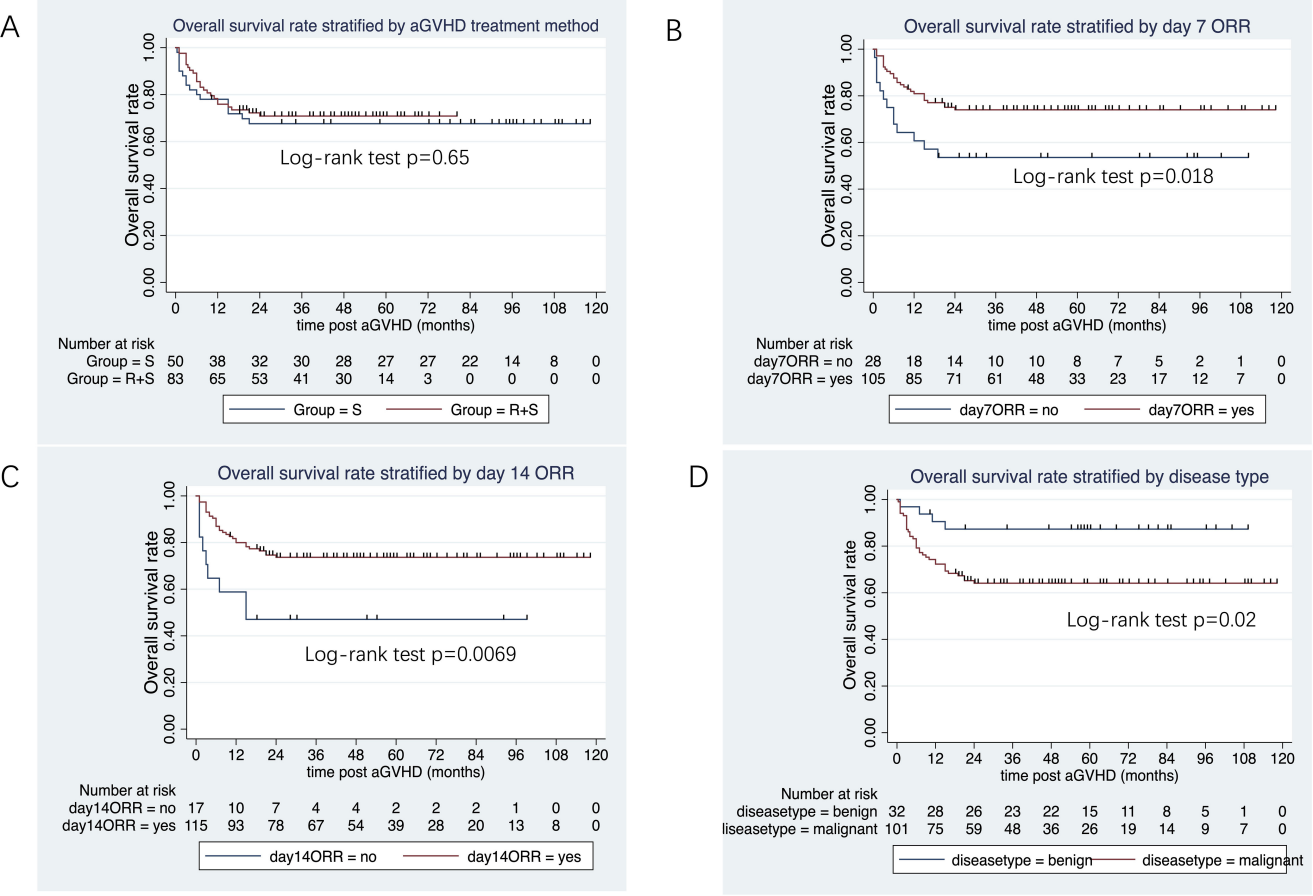
Overall survival rate stratified by aGVHD treatment, day 7 ORR, day 14 ORR and disease type. **(A)** Overall survival rate stratified by aGVHD treatment. **(B)** Overall survival rate stratified by day 7 ORR. **(C)** Overall survival rate stratified by day 14 ORR. **(D)** Overall survival rate stratified by disease type.

**Figure 3:**
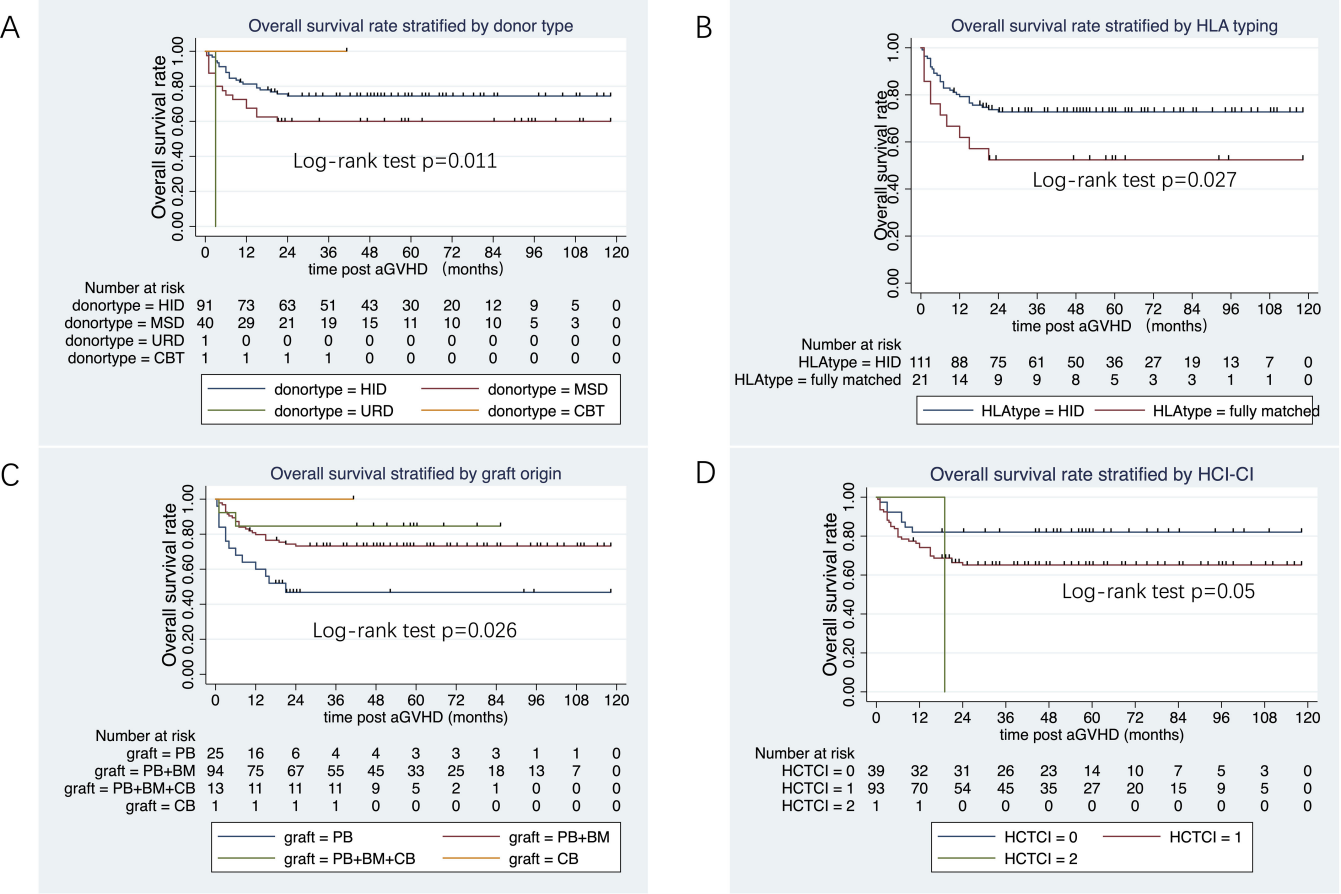
Overall survival rate stratified donor type, HLA typing, graft origin and HCT-CI. **(A)** Overall survival rate stratified by donor type. **(B)** Overall survival rate stratified by HLA. **(C)** Overall survival rate stratified by graft origin. **(D)** Overall survival rate stratified by HCT-CI.

**Figure 4:**
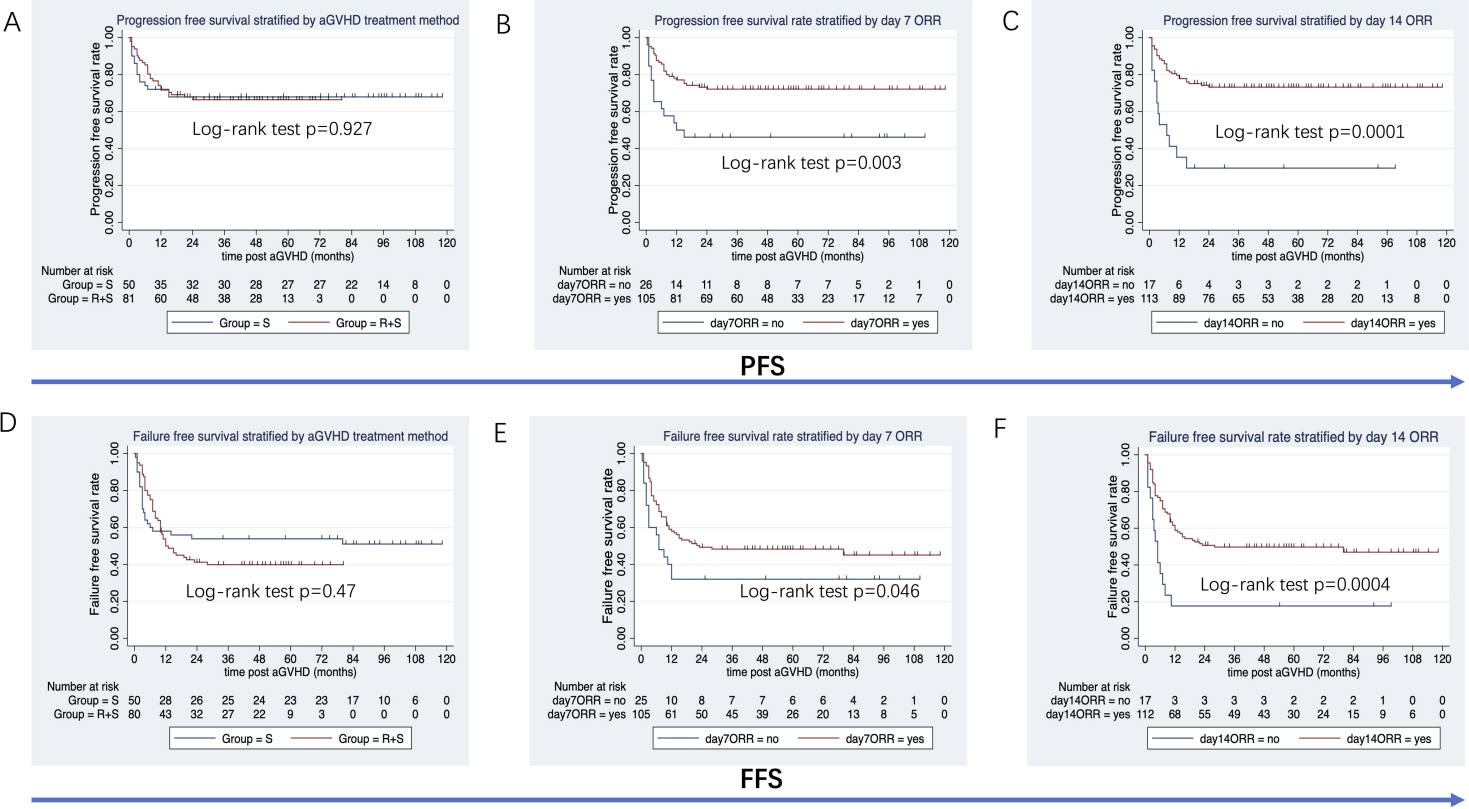
PFS and FFS stratified by aGVHD treatment, day 7 ORR and day 14 ORR. **(A)** PFS stratified by aGVHD treatment. **(B)** PFS stratified by day 7 ORR. **(C)** PFS stratified by day 14 ORR. **(D)** FFS stratified by aGVHD treatment. **(E)** FFS stratified by day 7 ORR. **(F)** FFS stratified by day 14 ORR.

**Figure 5:**
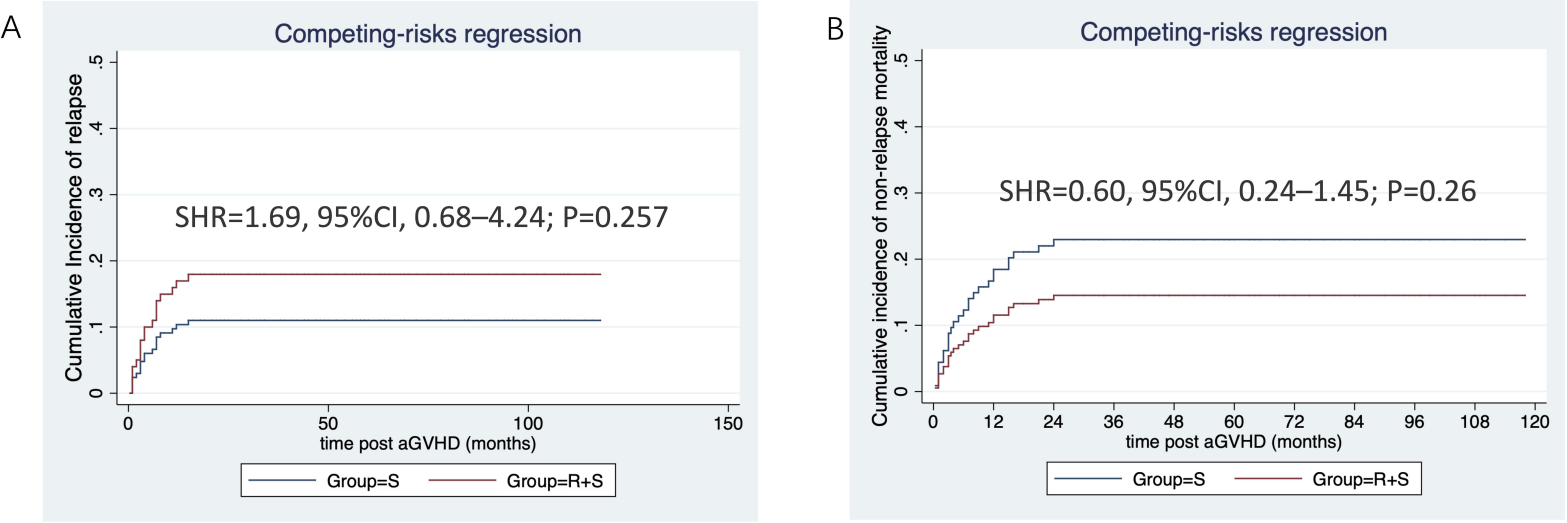
CIR and NRM stratified by aGVHD treatment. **(A)** Cumulative incidence of relapse stratified by aGVHD treatment. **(B)** Cumulative incidence of non-relapse mortality stratified by aGVHD treatment.

The median follow-up duration from aGVHD onset was 47 months (range: 1–80 months) in the ruxolitinib/steroids group and 90 months (range: 0.4–118 months) in steroid-only group. The 3-year OS rates were similar between two groups: 70.5% in the ruxolitinib/steroids group vs. 67.6% in the steroid-only group, both in Cox regression analysis (hazard ratio [HR]=0.86, 95% CI: 0.45–1.61, p=0.62) (**Table 3**) and Kaplan-Meier survival analysis (Log-rank test, p=0.65. **Figure 2A**). **Table 3** shows the univariate and multivariate analysis results for risk factors influencing OS. Univariate analysis revealed the following: (i) ORR on day 7 and day 14 had a significantly positive effect on OS (HR=0.45, 95% CI: 0.23–0.88, p=0.02) (HR=0.37, 95% CI: 0.17–0.78, p=0.009), confirmed by Kaplan-Meier survival analysis (**Figures 2B, C**). (ii) Malignant hematologic disease had a significantly negative effect on OS (HR=3.26, 95% CI: 1.16–9.17, p=0.02), also confirmed by Kaplan-Meier survival analysis (**Figure 2D**). (iii) Other factors-including donor type (CBT vs. HID vs. MSD vs. URD), HLA type (fully matched vs. haploidentical), stem cell source (CBT vs. PB+BM+CB vs. PB+BM vs. PB), and HCI-CI-influenced OS (**Table 3**, **Figure 3**). In the multivariate analysis, only day 14 ORR (HR=0.37, 95% CI: 0.16–0.88, p=0.02), aGVHD grade (HR=3.21, 95% CI, 0.96–10.6, p=0.05), and HCI-CI (HR=2.43, 95% CI, 0.99–5.99, p=0.05) influenced OS (**Table 3**). Additionally, we analyzed other major risk variables for OS (**Table 3**). Recipient sex, age at transplantation, donor sex, recipient-donor blood pair, aGVHD skin involvement, aGVHD GI involvement, and aGVHD liver involvement had no statistically significant effect on OS. Though the 1-year PFS rates were similar between the two groups-74% in the ruxolitinib/steroids group and 72% in the steroid-only group, as shown in Kaplan-Meier survival analysis (**Figure 4A**, log-rank test, p=0.927)—ORR on day 7 (**Figure 4B**, log-rank test, p=0.03) and day 14 (**Figure 4C**, log-rank test, p=0.001) demonstrated a significantly more favorable impact on PFS. The 1-year FFS rates were similar between the two groups, with 56.2% in the ruxolitinib/steroids group and 55.9% in the steroid-only group (**Figure 4D**, log-rank test, p=0.47). Reaching ORR on day 7 (**Figure 4E**, log-rank test, p=0.046) and day 14 (**Figure 4F**, log-rank test, p=0.0004) had a significantly more favorable effect on FFS. Both groups had comparable 2-year cumulative incidence rates of relapse (ruxolitinib/steroids: 18% vs. steroid-only: 11%; Subdistribution HR, SHR=1.69, 95% CI: 0.68–4.24, p=0.257; **Figure 5A**) and non-relapse mortality (ruxolitinib/steroids: 15% vs. steroid-only: 23%; SHR=0.60, 95% CI: 0.24–1.45, p=0.26; **Figure 5B**).

**Table 3:** Univariate and multivariate analysis of predictors for OS.

Factors	Values	OS	
		Univariate	Multivariate
		HR 95%CI p	HR 95%CI p
Group	(R+S vs S)	0.86 (0.45-1.61) 0.62	
Sex	(Male vs Female)	0.82 (0.44-1.52) 0.52	
Age at HSCT	(>60 vs ≤60)	1.33 (0.18-9.73) 0.78	
Disease type	(Malignant vs Benign)	3.26 (1.16-9.17) 0.02	
Donor Gender	(Female donor to Male recipient vs Others)	1.11 (0.53-2.33) 0.78	
Donor type	(CBT vs HID vs MSD vs URD)	0.52 (0.94-2.45) 0.08	
HLA type	(Fully match vs Haplo)	2.04 (0.99-4.17) 0.05	
Blood type	(Match vs Mismatch)	0.93 (0.49-1.74) 0.81	2.43 (0.99-5.99) 0.05
Graft origin	(CBTvsPB+BM+CBvsPB+BMvsPB)	0.43 (0.24-0.77) 0.004	
HCT-CI	(2 vs 1 vs 0)	2.17 (1.02-4.62) 0.04	
aGVHD grade	(IV vs III vs II vs I)	1.32 (0.93-1.87) 0.1	3.21 (0.96-10.6) 0.05
aGVHD skin involve	(Yes vs No)	0.91 (0.49-1.69) 0.77	
aGVHD GI involve	(Yes vs No)	1.39 (0.71-2.74) 0.33	
aGVHD liver involve	(Yes vs No)	1.57 (0.55-4.42) 0.39	
Day 3 ORR	(Yes vs No)	0.82 (0.44-1.54) 0.55	
Day 7 ORR	(Yes vs No)	0.45 (0.23-0.88) 0.02	
Day 14 ORR	(Yes vs No)	0.37 (0.17-0.78) 0.009	0.37 (0.16-0.88) 0.02
Day 28 ORR	(Yes vs No)	0.44 (0.15-1.24) 0.12	

R, ruxolitinib; S, steroid; HID, haplo-identical donor; MSD, matched sibling donor; URD, matched unrelated donor; CBT, cord blood transplantation; BM, bone marrow; PB, peripheral blood; CB, cord blood; HCT-CI, hematopoietic cell transplant-comorbidity index; aGVHD, acute graft versus host disease; ORR, overall response rate; 95%CI, 95% confidence interval.

### 3.4 Subgroup analysis

**Figure 6:**
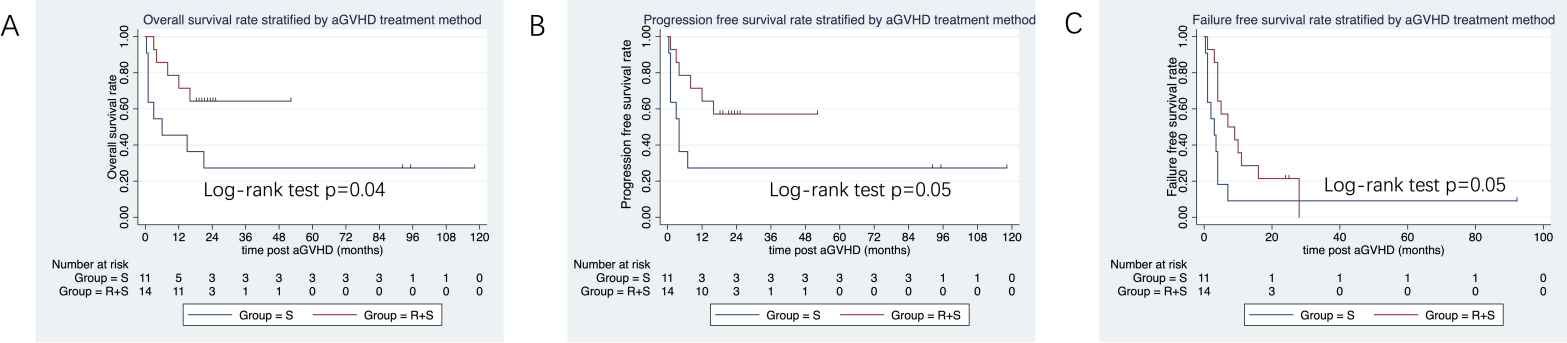
OS, PFS and FFS stratified by aGVHD treatment in patients who received peripheral blood (PB) grafts. **(A)** OS stratified by aGVHD treatment in patients with PB grafts. **(B)** PFS and FFS stratified by aGVHD treatment in patients with PB grafts. **(C)** FFS stratified by aGVHD treatment in patients with PB grafts.

In the subgroup of patients who received peripheral blood stem cell transplantation, the 3-year OS tended to be higher in the ruxolitinib/steroid combination group than in the steroid-only group (64.3% vs. 27.3%; log-rank test, p=0.046; **Figure 6A**). Similarly, the 3-year PFS was superior in the ruxolitinib/steroid group compared to the steroid-only group (57.1% vs. 27.3%; log-rank test, p=0.05; **Figure 6B**). Finally, the 2-year FFS was better in the ruxolitinib/steroid combination group than in the steroid-only group (21% vs. 9%; log-rank test, p=0.05; **Figure 6C**).

### 3.5 Safety and tolerance analysis

The median duration of ruxolitinib exposure was 70 days (range: 9–730 days) among the 83 patients, and none of the 83 patients discontinued ruxolitinib due to side effects, especially hepatotoxicity. No ruxolitinib toxicity related death was observed. As shown in **Table 4**, the most frequently observed AEs in the ruxolitinib/steroid group were neutropenia (32.5% vs. 12%, p=0.008), CMV infection (34.9% vs. 18%, p=0.036), and renal toxicity (20.4% vs. 6%, p=0.024). Other AEs, including thrombocytopenia, EBV infection, cardiac toxicity, nerve toxicity, electrolyte disturbance, blood glucose disturbance, blood lipid disturbance, and hypertension, were comparable between the two groups.

**Table 4:** Adverse events comparison (n=133).

AE	No. (%)		*p* value
	R+S (n=83)	S (n=50)	
Neutropenia	27 (32.5)	6 (12)	0.008
Thrombocytopenia	29 (34.9)	15 (30)	0.558
CMV infection	29 (34.9)	9 (18)	0.036
EBV infection	23 (27.7)	12 (24.4)	0.685
Renal toxicity	17 (20.4)	3 (6)	0.024
Cardiac toxicity	2 (2.4)	1 (2)	0.878
Digestive reaction	4 (4.8)	8 (16)	0.029
Nerve toxicity	2 (2.4)	1 (2)	0.878
Electrolyte disturbance	27 (32.5)	20 (40)	0.383
Blood glucose disturbance	15 (18)	6 (12)	0.352
Blood lipid disturbance.	31 (37.3)	11 (13.3)	0.065
Hypertension	4 (4.8)	1 (2)	0.115

R, ruxolitinib; S, steroid; AE, adverse events.

## 4 Discussion

With an incidence of 30–60% and a mortality rate of 15–30%, aGVHD is a serious complication of allo-HSCT (4, 29, 30). Systemic steroids are the first-line treatment for aGVHD grades II–IV, according to the European Group for Blood and Marrow Transplantation (EBMT). Unfortunately, 50% of the patients with aGVHD relapse or do not respond to steroids (31). According to the findings of the REACH1 and REACH2 trials, which assessed ruxolitinib as a second-line treatment, the overall response on day 28 was higher in the ruxolitinib group than in the control group, which included therapies such as ATG, extracorporeal photopheresis, mesenchymal stromal cells, low-dose methotrexate, mycophenolate mofetil, mammalian target of rapamycin (mTOR) inhibitor (everolimus or sirolimus), etanercept, and infliximab. Ruxolitinib is currently the only medication authorized for the treatment of SR-aGVHD (22, 32, 33). Despite the recognized benefits of first-line treatment for aGVHD, prolonged steroid therapy can lead to serious adverse effects, such as weight gain, hyperglycemia, diabetes mellitus, adrenal suppression, osteoporosis, dermatological changes, cardiovascular complications, cataracts, glaucoma, peptic ulcers, myopathy, increased propensity for infections, and neuropsychiatric disorders (34). Additionally, half of patients do not respond to steroids. Therefore, the first-line treatment of aGVHD is an unmet need in clinical practice, and novel active first-line treatments are urgent for improving efficiency and decreasing side effects in newly diagnosed patients with aGVHD. Liu et al. (23) designed the first prospective, randomized controlled trial to show that combining ruxolitinib (5mg/day) and methylprednisolone (1mg/kg/day) is a superior first-line therapy for intermediate- and high-risk aGVHD compared to the standard 2 mg/kg/day methylprednisolone regimen. This novel approach significantly improved ORRs on days 28 and 56, resulted in a more durable response at 6 months, and led to better failure-free survival than corticosteroid monotherapy. Moreover, this first-line therapy was well-tolerated and reduced exposure to steroids, opening a new chapter for the treatment of aGVHD. In real-world scenarios, the efficacy and safety of ruxolitinib and steroid combination therapy in the first-line treatment of aGVHD are still unclear, and we performed this retrospective study to focus on this issue.

We designed this study based on following reasons: 1) Considering the significant morbidity and mortality associated with aGVHD, there is an unmet need for effective and evidence based new options for the management of aGVHD. 2) Our center has noticed that the clinical research results related to the treatment of SR-GVHD with ruxolitinib internationally are encouraging. Spoerl et al. (20) reported that 6 SR-GVHD patients responded with respect to clinical GVHD symptoms and serum levels of proinflammatory cytokines after treatment with ruxolitinib in 2014; Zeiser et al. (21) reported on a multicenter clinical study in Europe and USA, which retrospectively analyzed the effectiveness of ruxolitinib in treating SR-GVHD in 2015. 3) Ruxolitinib was approved and accessible in China in March 2017. Therefore, we speculate that the first-line treatment of ruxolitinib/steroid may reduce the occurrence of SR-GVHD and increase the ORR rate. Our center first integrates ruxolitinib in the treatment strategy of aGVHD in December 2017.

In our study, ruxolitinib was administered as first-line therapy at a dosage of 10-15 mg/day combined with methylprednisolone (0.5–2 mg/kg/day). The primary endpoint, ORR on days 7 and 14, was significantly higher in the ruxolitinib/steroid combination group than that in the steroid-only group. However, ORR on day 28 was similar between the two cohorts. The ORR response in our study appeared to be achieved earlier than in Liu’s prospective trial (23). This phenomenon might be partially explained by the relatively high ruxolitinib dose used in our study. Although no statistical differences in OS, PFS, and FFS were observed between the two groups, patients with aGVHD who achieved early ORR on day 7 or 14 had superior OS, PFS, and FFS. We hypothesize that the ruxolitinib/steroid combination cohort could indirectly influence OS, PFS, and FFS by improving the early ORR on days 7 and 14. Notably, successful control of aGVHD does not always correlate with enhanced survival. For instance, Robert Zeiser (32) reported a higher ORR on day 28 in the ruxolitinib group than in the control group among patients with SR-aGVHD, but no significant difference in the 18-month OS. Similarly, in the trial by Liu et al, the 18-month OS rates were comparable between groups, at 75.4% in the RUX/steroid group and 70.5% in the steroid-only group (p=0.734). The authors mentioned that, in GVHD treatment trials, discrepancies between response and survival outcomes were likely influenced by factors such as infections, regimen-related toxicity, relapse of malignancy, and underlying conditions unrelated to GVHD (23). Interestingly, in our subgroup of patients who received peripheral blood stem cell transplantation, the 3-year OS, PFS, and 2-year FFS were superior in the ruxolitinib/steroid group than in the steroid-only group.

The most frequently reported adverse effects of ruxolitinib include infectious complications and cytopenia. In our study, neutropenia and CMV infection were more common in the ruxolitinib/steroid group, which could be managed with granulocyte colony-stimulating factor (G-CSF) and antiviral agents (ganciclovir/foscarnet). Other AEs, including thrombocytopenia, EBV infection, cardiac toxicity, nerve toxicity, electrolyte disturbance, blood glucose disturbance, blood lipid disturbance, and hypertension, were comparable between the two groups. This indicates that adding ruxolitinib to first-line steroid treatment for aGVHD does not increase severe AEs, and they remain controllable.

Real-world data play an important role in generating evidence complementary to conventional randomized controlled trials (RCT) and in improving clinical trial designs. Real-world studies have collected data from patients receiving clinical care in routine practice and provided more authentic evidence. In line with other real-world studies, one of the key limitations of our study is that the intervention of interest was not randomly assigned, which could have resulted in biased associations between the treatment and outcomes of interest. It is vital to understand the strengths and limitations of real-world and RCT evidence to implement a framework in which they can be used complementarily to create a robust evidence base for treatment decision-making. In conclusion, in our real-world clinical setting, first-line use of methylprednisolone combined with ruxolitinib proved superior to conventional methylprednisolone monotherapy for aGVHD, as demonstrated by the significantly improved overall response.

## Data Availability

The raw data supporting the conclusions of this article will be made available by the authors, without undue reservation.
